# A Reappraisal of the Mechanism by Which Plant Sterols Promote Neutral Sterol Loss in Mice

**DOI:** 10.1371/journal.pone.0021576

**Published:** 2011-06-30

**Authors:** Gemma Brufau, Folkert Kuipers, Yuguang Lin, Elke A. Trautwein, Albert K. Groen

**Affiliations:** 1 Department of Pediatrics, Center for Liver, Digestive and Metabolic Diseases, University Medical Center Groningen, University of Groningen, Groningen, The Netherlands; 2 Departments of Pediatrics and Laboratory Medicine, Center for Liver, Digestive and Metabolic Diseases, University Medical Center Groningen, University of Groningen, Groningen, The Netherlands; 3 Unilever R&D, Vlaardingen, The Netherlands; University of Padova, Medical School, Italy

## Abstract

Dietary plant sterols (PS) reduce serum total and LDL-cholesterol in hyperlipidemic animal models and in humans. This hypocholesterolemic effect is generally ascribed to inhibition of cholesterol absorption. However, whether this effect fully explains the reported strong induction of neutral sterol excretion upon plant sterol feeding is not known. Recent data demonstrate that the intestine directly mediates plasma cholesterol excretion into feces, i.e., without involvement of the hepato-biliary route.

**Objective:**

Aim of this study was to determine whether stimulation of fecal neutral sterol loss during PS feeding is (partly) explained by increased intestinal cholesterol excretion and to assess the role of the cholesterol transporter Abcg5/Abcg8 herein.

**Methods and Results:**

Wild-type mice were fed a control diet or diets enriched with increasing amounts of PS (1%, 2%, 4% or 8%, wt/wt) for two weeks. In addition, *Abcg5^-/-^* mice were fed either control or 8% PS diet. PS feeding resulted in a dose-dependent decrease of fractional cholesterol absorption (∼2–7-fold reduction) in wild-type mice and ∼80% reduction in Abcg5^-/-^ mice. Furthermore, PS feeding led to a strong, dose-independent induction of neutral sterol excretion (3.4-fold in wild-types and 2.7-fold in *Abcg5^-/-^* mice) without changes in biliary cholesterol secretion. It was calculated that PS feeding stimulated intestinal cholesterol excretion by ∼500% in wild-type mice and by ∼250% in *Abcg5^-/-^*.

**Conclusions:**

Our data indicate that in mice the cholesterol-lowering effects of PS are to a large extent attributable to stimulation of intestinal, non-bile derived, cholesterol excretion. The Abcg5/Abcg8 heterodimer is involved in facilitating this PS-induced flux of cholesterol.

## Introduction

Addition of plant sterols to the diet reduces serum total and LDL-cholesterol concentrations in normolipidemic and hypercholesterolemic subjects without affecting serum HDL-cholesterol levels [1].

The structural similarity between plant sterols and cholesterol presumably under-lies their effects on cholesterol metabolism. Plant sterols compete with cholesterol for incorporation into mixed micelles present in the upper small intestine, which contributes to a decreased fractional cholesterol absorption and a consequent increase in the amount of cholesterol excreted as neutral sterol into feces [2]. In addition, plant sterols may influence expression and/or activity of proteins involved in cholesterol metabolism, both in enterocytes and hepatocytes [for review see [3]]. Several in vivo and in vitro studies have shown that plant sterols are able to act as LXR ligands leading to induction of cholesterol transporter gene expression [4]-[6], although this could not be confirmed in other studies [7]. It has also been proposed that plant sterols may reduce plasma cholesterol levels via LXR-independent mechanisms [3]. Proposed modes of action involve (i) reduction of HMG-CoA reductase activity, i.e., interfering with cholesterol synthesis [8], (ii) decreasing ACAT activity, hence reduction of cholesterol esterfication needed for incorporation into chylomicrons [9] or (iii) interference with cholesterol trafficking within the enterocytes since plant sterols may alter expression of genes encoding proteins of the ANX family that are involved in regulation of membrane properties [10].

Recently, the existence of a route for cholesterol removal from the body that does not involve the hepato-biliary system has been demonstrated [11]. This route has been called transintestinal cholesterol excretion (TICE). The existence of TICE could be deduced from several observations. For instance, Kruit et al. reported that in Mdr2-/- mice, which have an almost completely abolished biliary cholesterol secretion, fecal netural sterol output did not differ from that in wild-type mice [12]. Moreover, challenging these mice with a LXR agonist resulted in a 2-fold increase in fecal neutral sterols without any stimulation of biliary cholesterol secretion [12]. Furthermore, overexpression of hepatic Niemann-Pick C1 Like 1 (Npc1l1) in liver resulted in 10- to 20-fold lower biliary cholesterol concentrations in mice [13], whereas fecal neutral sterol excretion did not differ from control values in these animals [14]. Recently, Temel and colleagues determined the flux of macrophage reverse cholesterol transport in two different mouse models of suppressed biliary cholesterol secretion, i.e., bile diverted mice and Npc1l1 overexpression. In both models, biliary cholesterol secretion was dramatically reduced or even abolished, whereas fecal neutral sterol mass remained unaffected [15]. In an earlier study from our laboratory, Plösch et al. reported that mice fed plant sterols showed a 5-fold induction in fecal neutral sterol output while dietary cholesterol intake and biliary cholesterol output were unchanged [16]. Even when fractional intestinal absorption of cholesterol would be completely blocked, i.e., equal 0%, a substantial fraction of cholesterol excretion remains that cannot be explained by the commonly accepted mechanisms. Thus, it seems conceivable that plant sterol feeding induces TICE but this has so far not been demonstrated.

To date, limited data is available about the regulation of TICE, the source of the cholesterol excreted or the lipoproteins and transporters involved. Although HDL seems the most likely lipoprotein involved, data coming from different laboratories indicate that this is unlikely. Abca1-/- mice are characterized by absence of HDL yet show no decrease in fecal neutral sterol excretion [17]–[19]. Therefore, it appears that another, yet undentified lipoprotein must be involved. Similarly, a number of proteins have been proposed as candidates to facilitate the cholesterol efflux from blood to the intestinal lumen [11]. Several attempts have been made to unravel the role of the heterodimer Abcg5/Abcg8 in TICE [20]–[25] but, so far, the results have not been conclusive.

The aim of this study was to determine whether plant sterol feeding stimulates the non-biliary route of cholesterol excretion, i.e., direct secretion of cholesterol by the intestine. For this purpose, we quantified kinetic parameters of cholesterol metabolism in wild-type mice fed increasing amounts of plant sterols (0%, 1%, 2%, 4% and 8%; wt/wt). To determine the role of the Abcg5/Abcg8 heterodimer in the plant sterol-induced stimulation of fecal neutral sterol output, we performed similar experiments in the Abcg5-deficient mouse, an animal model for sitosterolemia [24].

## Results

### Effects of plant sterols on plasma and hepatic parameters

Since it has recently been published that there is a dose-dependency in the cholesterol-lowering efficiency of dietary plant sterols (up to 9g/d) in humans [29], we studied cholesterol kinetics in wild-type mice fed increasing amounts of plant sterols (0%, 1%, 2%, 4% and 8%, wt/wt). No differences in body weights were seen between the respective groups of animals, although food intake by mice fed 8% plant sterols was higher compared to mice on control diet. Plant sterol feeding increased fecal mass (dry weight) in all groups (**Table 1**). Total plasma cholesterol was reduced in wild-type or in *Abcg5^-/-^* mice fed 8% plant sterol diet (**Table 2**). No differences due to the dietary interventions were found in any of the hepatic parameters (**Table 3**). No changes in biliary cholesterol, phospholipid or bile acid excretion rates were seen in wild-type mice or in *Abcg5^-/-^* mice fed plant sterols: only on the highest dose of plant sterols (8%), wild-type mice did show lower biliary cholesterol excretion rates than mice fed control diet (**Table 4**).

**Table 1:** Basal parameters in wild-type and Abcg5^-/-^ mice fed either control or plant sterol diet.

	Wild-type mice					Abcg5^-/-^ mice	
	0% PS	1% PS	2% PS	4% PS	8% PS	0% PS	8% PS
**Basal parameters**							
**Body weight (g)**	28.1±2.6	29.6±2.8	31.0±2.6	30.2±2.2	28.5±1.9	26.6±2.7	26.9±3.1
**Food intake (g/d)**	3.0±0.5	2.2±1.1*	3.1±0.5	3.0±0.4	3.7±0.5*	3.3±0.6	5.1±1.4*
**Fecal output (g/d)**	0.38±0.03	0.41±0.02*	0.49±0.05*	0.50±0.05*	0.68±0.10*	0.42±0.01^#^	0.67±0.09*

Values represent average ± SD. n = 4–11.

*P<0.05 control *vs* plant sterol fed wild-type mice; #*P*<0.05 control *vs* plant sterol fed Abcg5^-/-^ mice.

**Table 2:** Plasma lipid concentrations in wild-type and Abcg5^-/-^ mice fed either control or plant sterol diet.

	Wild-type mice					Abcg5^-/-^ mice	
	0% PS	1% PS	2% PS	4% PS	8% PS	0% PS	8% PS
**Triglycerides (mM)**	0.78±0.27	0.71±0.16	0.60±0.10	0.58±0.12*	0.68±0.16	1.02±0.33	1.22±0.47^#^
**Cholesterol (mM)**	4.1±0.2	3.6±0.5*	4.0±0.3	3.7±0.4*	3.1±0.3*	2.0±0.2^#^	1.5±0.3^#^
**β-sitosterol (µM)**	7.8±2.7	32.6±12.8*	34.20±9.3*	28.4±8.4*	36.5±2.7*	447±57.0^#^	1604±399* ^#^
**Campesterol (µM)**	18.0±4.6	41.7±11.8*	38.9±11.0*	33.0±5.9*	47.6±5.0*	113±13.6^#^	439±129* ^#^

Values represent average ± SD. n = 4–11.

*P<0.05 control *vs* plant sterol fed wild-type mice.

#*P*<0.05 control *vs* plant sterol fed Abcg5^-/-^ mice.

**Table 3:** Hepatic lipid concentrations in wild-type and Abcg5^-/-^ mice fed either control or plant sterol diet.

	Wild-type mice					Abcg5^-/-^ mice	
	0% PS	1% PS	2% PS	4% PS	8% PS	0% PS	8% PS
**Liver**							
**Liver weight (%BW)**	4.4±0.4	4.3±0.4	4.6±0.5	4.3±0.4	4.4±0.2	5.4±0.5^#^	6.0±1.0^#^
**Phospholipids (µmol/g)**	4.1±0.6	3.7±0.2*	3.7±0.2*	3.8±0.4	4.4±0.2	4.3±0.2	4.6±0.2
**Triglycerides (µmol/g)**	18.4±7.3	20.1±7.0	29.3±8.3*	18.8±7.3	23.8±15.1	15.5±9.0	9.0±7.8
**Cholesterol (µmol/g)**	3.70±.05	3.43±0.58	3.27±0.58	3.10±1.84	2.85±1.60	2.73±1.22	2.42±1.11
**β-sitosterol (nmol/g)**	8.9±6.5	29.0±10.2*	28.4±5.5*	25.7±4.8*	40.8±9.4*	414.9±39.4^#^	1110.1±654.9* ^#^
**Campesterol (nmol/g)**	21.8±9.8	41.1±11.0*	38.3±8.1*	38.4±2.6*	65.6±18.1*	151.6±16.5^#^	420.3±202.3* ^#^

Values represent average ± SD. n = –11.

*P<0.05 control *vs* plant sterol fed wild-type mice; #*P*<0.05 control *vs* plant sterol fed Abcg5^-/-^ mice.

**Table 4:** Hepatobiliary lipid output of wild-type or *Abcg5^-/-^* mice fed control or plant sterol diet.

	Wild-type mice					Abcg5^-/-^ mice	
	0% PS	1% PS	2% PS	4% PS	8% PS	0% PS	8% PS
**Bile flow (µl/100 g/d)**	7.4±1.3	6.5±1.0	7.0±.2	6.9±0.9	5.1±1.1*	8.0±2.4	9.1±1.3^#^
**Cholesterol (µmol/100 g/d)**	3.9±1.5	3.5±0.5	3.9±1.8	3.9±0.8	2.1±0.6*	1.9±0.7^#^	2.2±0.3
**Phospholipid (µmol/100 g/d)**	35.3±9.2	31.5±7.5	29.9±5.1	30.0±9.0	28.3±7.5	19.3±6.7^#^	17.2±10.4
**Bile acid (µmol/100 g/d)**	305±146	294±123	344±129	295±134	178±78	245±120	210±78

Values represent average ± SD. n = 4–11.

*P<0.05 control *vs* plant sterol fed wild-type mice; #*P*<0.05 control *vs* plant sterol fed Abcg5^-/-^ mice.

### Fractional cholesterol absorption decreases in a dose-dependent manner upon plant sterol feeding

**Figure 1:**
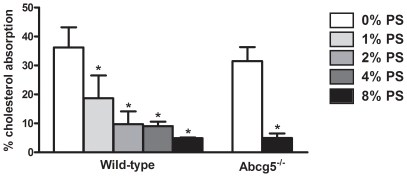
Fractional absorption of cholesterol in wild-type and *Abcg5^-/-^* mice fed control or diet supplemented with varying doses of plant sterols. Values represent average ± SD. n = 4–11. *P<0.05 control *vs* plant sterol fed wild-type mice; #*P*<0.05 control *vs* plant sterol fed Abcg5^-/-^ mice.

As expected, increasing the intake of dietary plant sterols resulted in a decreased fractional cholesterol absorption in wild-type mice (from 40% in control diet to 5% on 8% plant sterols; p<0.001) and in *Abcg5* ^-/-^ mice (from 32% to 5%; p<0.001; **Figure 1**).

### Plant sterol feeding increases fecal cholesterol output, partly via Abcg5

**Figure 2:**
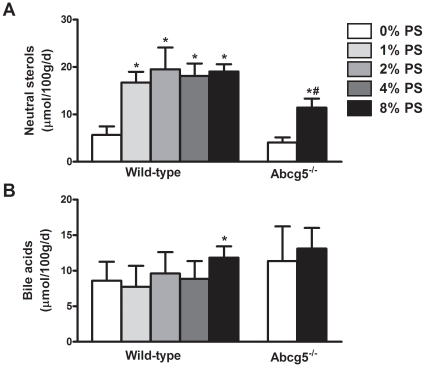
Fecal loss of neutral sterols and bile acids in wild-type and *Abcg5^-/-^* mice fed either control or diet enriched with varying doses of plant sterols. Values represent average ± SD. n = 4–11. *P<0.05 control *vs* plant sterol fed wild-type mice; #*P*<0.05 control *vs* plant sterol fed Abcg5^-/-^ mice.

Interestingly, the effect of increasing plant sterol intake on cholesterol absorption was not mirrored in fecal neutral sterol excretion. For all plant sterol-enriched diets, two weeks of feeding resulted in 3.4-fold induction in fecal neutral sterol excretion in wild-type mice. In *Abcg5^-/-^* mice, plant sterol feeding also resulted in an increased fecal neutral sterol output, although the effect was more moderate in this case (**Figure 2A**). Plant sterol feeding slightly enhanced fecal bile acid excretion in wild-type mice fed the highest concentration of plant sterols used. In *Abcg5^-/-^* mice the same trend was observed, but this effect did not reach statistical significance (**Figure 2B**).

### Non-biliary cholesterol excretion is enhanced after plant sterol feeding

To test whether plant sterol feeding stimulates the non-biliary route of cholesterol excretion, i.e., direct intestinal secretion, we determined intestinal cholesterol balance. This balance is defined by the sum of daily cholesterol intake and the estimated amount of biliary cholesterol excreted per day minus the amount absorbed per day, as calculated from the fractional cholesterol absorption. Subtraction of this amount from the amount of neutral sterols excreted into the feces per day, yields the value for non-biliary cholesterol excretion.

**Figure 3:**
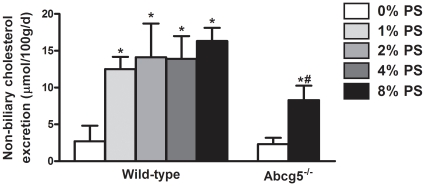
Non-biliary cholesterol excretion in wild-type and *Abcg5^-/-^* mice fed mice fed either control or diet enriched with varying doses of plant sterols. Values represent average ± SD. n = 4–11. *P<0.05 control *vs* plant sterol fed wild-type mice; #*P*<0.05 control *vs* plant sterol fed Abcg5^-/-^ mice.

Plant sterol supplementation led to a 6-fold induction of the non-biliary cholesterol excretion in wild-type mice (2.70±2.13 µmol/100 g/day in control diet-fed mice *vs* 16.3±.8 µmol//100 g/day in 8% plant sterol-fed mice; p<0.01). Interestingly, 8% plant sterol feeding resulted in an increased non-biliary cholesterol excretion also in *Abcg5^-/-^* mice. However, this increase was significantly less than that observed in wild-type mice (∼3.5-fold) (**Figure 3**).

### mRNA levels and protein expression

**Figure 4:**
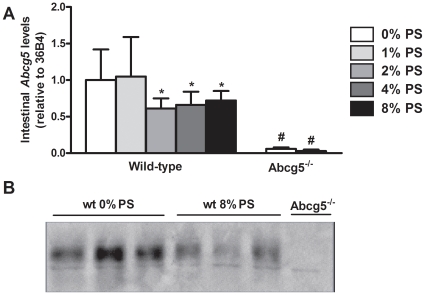
mRNA levels and protein expression of Abcg5 in wild-type and *Abcg5^-/-^* mice fed mice fed either control or diet enriched with plant sterols. Values represent average ± SD. n = 4–11. *P<0.05 control *vs* plant sterol fed wild-type mice; #*P*<0.05 control *vs* plant sterol fed Abcg5^-/-^ mice.

In order to further delineate the potential role of the intestinal Abcg5/Abcg8 heterodimeric transporter in the process described, we measured *Abcg5* mRNA levels and protein expression in the intestine of wild-type mice fed either control diet or diet supplemented with plant sterols. Surprisingly, plant sterol feeding resulted in down-regulation of *Abcg5* mRNA expression (**Fig. 4A**). Similarly, also Abcg5 protein expression was lower in animals fed plant sterols compared to those fed control diet (**Fig. 4B**). In line with these results, mRNA levels of *Abcg8* and *Abca1* were lower in the small intestines of mice fed plant sterols compared to those of control mice. Conversely, no differences were found in the expression of any of these genes in the liver (**Table S2**). As expected, plant sterol feeding resulted in increased cholesterol synthesis at least in wild-type mice, as deduced from induction in *Hmgcr* mRNA levels in liver and intestine, in order to compensate for the cholesterol loss in the feces (**Table S2**).

## Discussion

This study demonstrates, for the first time, that the plant sterol-induced increase in fecal neutral sterol excretion in mice is mainly caused by an increase of direct intestinal cholesterol excretion, i.e., bypassing the “classical” hepato-biliary route, rather than by inhibition of cholesterol absorption. Furthermore, our data indicate a role, but a non-exclusive one, for the Abcg5/Abcg8 transporter in maintenance of this novel pathway mediating cholesterol excretion.

In the literature, there is a general consensus that plant sterols reduce plasma cholesterol in humans because of their ability to displace cholesterol from the mixed micelles in the upper small intestine, thereby reducing the efficiency of cholesterol absorption [2]. However, in addition to this “micellar hypothesis”, alternative mechanisms have been proposed [3]. For instance, it has been reported that plant sterols are able to activate the nuclear receptor LXR, thereby up-regulating several ABC transporters such as ABCA1, ABCG5 and ABCG8 leading to changes in cholesterol transport [4]–[6]. However, in our study expression levels of these transporters were not changed or even decreased by plant sterol feeding, in agreement with a previous study [7]. The main finding of the current study is that the excretion of cholesterol via a non-biliary route is the predominant mechanism by which plant sterols increase fecal neutral sterol excretion in mice. Because wild-type mice carry their plasma cholesterol mainly in HDL, unlike humans, plant sterol feeding does not substantially reduce total plasma cholesterol in this species. Because non-biliary cholesterol excretion appears to be an important pathway also in humans (unpublished data), it may well be that the LDL-cholesterol lowering capacities of plant sterols in humans are, to a large extent, attributable to stimulation of TICE.

We estimated cholesterol excretion via the non-biliary route by performing a careful cholesterol balance analysis. As anticipated, plant sterol supplementation resulted in a 3.4-fold increase in the neutral sterol output, while dietary cholesterol intake and biliary cholesterol excretion remained essentially unchanged. Taking into account the fraction of cholesterol that is absorbed, the flux of non-biliary cholesterol excreted into the feces under the different dietary conditions could be estimated. Plant sterol feeding resulted in a 6-fold induction of this flux in wild-type mice. Interestingly, this non-biliary route showed a higher sensitivity towards the actions of plant sterols than the absorption pathway. The lowest level of plant sterol supplementation (1%) resulted already in a maximal stimulation of fecal neutral sterol excretion and of non-biliary cholesterol excretion, in contrast to the “dose-dependent” pattern that was found for cholesterol (re)absorption (Fig 1, Fig. 2 and Fig. 3). This implicates that stimulation of intestinal cholesterol excretion constitutes a major mechanism to explain the cholesterol-lowering properties of plant sterols. Similar results were found in a study in which wild-type mice were fed a diet supplemented with plant sterols combined with cholesterol [16]. The authors reported an increase in fecal neutral sterols that could not be explained by the decreased fractional absorption after plant sterol feeding. However, in this particular study the contribution of the non-biliary route to fecal excretion of neutral sterols was not estimated.

In principle, cholesterol excreted via the non-biliary route can originate from enterocytes either as component of shedded cells or upon secretion of locally synthesized cholesterol. Alternatively, plasma-derived cholesterol can be transported directly through the intestine, a pathway called transintestinal cholesterol excretion (TICE). In this study, we were formally not able to distinguish between the different sources. Nevertheless, in previous studies, it has been estimated that the contribution of cholesterol originating from cell shedding ranges between 10 and 30% in wild-type mice [20], [25], [30]. In this study we show that the non-biliary route is strongly decreased in Abcg5^-/-^ mice. One might argue that this is not caused by a direct effect via abrogation of the activity of the sterol transporter but to a secondary effect on the turnover of intestinal cells. In an earlier study [20] we have estimated the contribution of cell shedding to fecal neutral excretion in Abcg5^-/-^ mice and their wild type litter mates. No difference was found indicating that the inihibition of non-biliary cholesterol secretion in Abcg5^-/-^ mice was not due to secondary effects on intestinal cell turnover. Furthermore, the contribution of intestine-derived *de novo* synthesized cholesterol to fecal neutral sterols is small in mice [20]. Although intestinal mRNA levels of *Hmgcr* were slightly induced by plant sterol feeding, we consider it unlikely that enhanced *de novo* synthesis provides an important source of fecal neutral sterols. Therefore, we hypothesize that the majority of the neutral sterols has actually been excreted *via* TICE.

The molecular mechanism of TICE has not yet been elucidated. Several transporters have been postulated to be involved in the efflux of cholesterol from blood into the intestinal lumen [11], but with exception of the heterodimer Abcg5/Abcg8 no evidence for transporter involvement has been reported. To address the possible role of Abcg5/Abcg8 in the plant sterol-induced increase in neutral sterol output, we performed experiments in Abcg5-deficient mice. Under the conditions used in this study, plant sterol feeding resulted in a 2.7-fold increase of fecal excretion in neutral sterols in *Abcg5^-/-^* mice, compared to a more than 3.4-fold induction in wild-type mice. This difference between genotypes is remarkable since the biliary cholesterol excretion and the cholesterol (re)absorption were similar between groups fed 8% plant sterols. Next, we estimated the flux of non-biliary cholesterol excretion in *Abcg5^-/-^* mice fed plant sterols. Plant sterol feeding increased the non-biliary cholesterol flux in *Abcg5^-/-^* mice (∼3.5-fold), but this increase was half of that observed in wild-type mice (∼6-fold induction). This data clearly indicates that the non-biliary cholesterol flux can be mediated via at least two pathways, one of them dependent on the activity of Abcg5/Abcg8. This role of dietary plant sterols on intestinal cholesterol transport is interesting in light of earlier studies in Abcg5/Abcg8 knock-out mice. No decrease in fecal neutral sterol excretion [24] or only a mild effect [23], [31] was observed in single knock-out (*Abcg5^-/-^* and/or *Abcg8^-/-^*) mice whereas the double knock-out did show a decrease in neutral sterol excretion [32]. Our group recently reported a decrease (∼20%) of neutral sterol excretion in chow-fed *Abcg5^-/-^* mice, which was paralleled by a 79% reduction in TICE compared to their wild-type littermates [20]. Although at first sight these results seem conflicting, it should be noted that in previous studies mostly chow diets were used that are known to be rich in plant sterols. In contrast, in the present study we used a semi-synthetic diet free of plant sterols as reference. Although our data clearly demonstrate a role for Abcg5/Abcg8 in non-biliary cholesterol excretion, we also must conclude that the heterodimer probably is not rate-controlling in this pathway. Despite the observed two-fold increase in flux through the Abcg5/Abcg8-mediated part of the pathway both mRNA levels and protein expression of Abcg5 decreased in the small intestine upon plant sterol feeding. We have not succeeded in visualizing the localization of the proteins in the enterocytes. Therefore, our data may not reflect the actual activity of this transporter at the apical membrane of the enterocytes. Further work is required to elucidate the exact role of Abcg5/Abcg8 in transintestinal cholesterol excretion.

Several attempts have been made to unravel the molecular regulation of TICE [20], [33], [34]. Van der Velde and colleagues reported that high-fat feeding stimulates TICE two-fold, whereas high-cholesterol feeding has no effect [33]. According to these authors, the effects observed under high-fat feeding may be due to activation of PPARδ as a result of fatty acid influx into the enterocytes [34]. In addition, LXR has also been suggested as a potential candidate in the regulation of TICE since mice fed an LXR agonist showed a 88% induction of TICE [20]. Some authors have reported that plant sterols are able to act as ligands of LXR [4]-[6], although others could not confirm these effects [3]. We have measured some LXR target genes in order to evaluate the potential LXR-activating effects of plant sterols in our study. Plant sterols did not affect or even down-regulated LXR target genes, indicating that stimulation of the non-biliary route of cholesterol excretion after plant sterol feeding is not LXR-mediated.

In conclusion, we show for the first time that plant sterol feeding results in stimulation of cholesterol excretion *via* the non-biliary route. In addition, using a knock-out model, we demonstrated that, under conditions in which this flux is stimulated by dietary plant sterols, the heterodimer Abcg5/Abcg8 mediates part of the increased cholesterol flux. Finally, the fact that most of the cholesterol found in feces is excreted *via* the non-biliary route upon plant sterol feeding in mice indicates that this route may also be important for the cholesterol-lowering properties of plant sterols in humans.

## Materials and Methods

### Animal experiments

Male *Abcg5^-/-^* mice and their wild-type littermates were kept in a light- and temperature-controlled environment. From weaning on, all mice were fed a purified diet free of plant sterols (Arie Blok, Woerden, The Netherlands). At three months of age, animals were matched for body weight and assigned to one of the treatment groups. Subsequently, wild-type mice were fed either the same purified diet (control diet; **table S1**) or the same purified diet supplemented with plant sterols (1%, 2%, 4% or 8%, wt/wt) for two weeks. *Abcg5^-/-^* mice received the control diet or the 8% plant sterol supplemented diet for two weeks. During the whole experiment, mice received food and water *ad libitum*. All experiments were approved by the local Ethical Committee for Animal Experiments of the University of Groningen.

### Experimental procedures

Cholesterol fluxes were measured as described previously [20], with some modifications. Briefly, at day 0, mice received an intravenous dose of 0.3 mg (0.73 µmol) cholesterol-D_7_ dissolved in Intralipid (20%, Fresenius Kabi, Den Bosch, The Netherlands) and an oral dose of 0.6 mg (1.535 µmol) cholesterol-D_5_ dissolved in medium-chain triglyceride oil. Blood spots were collected from the tail on filter paper (Schleicher & Schuell No2992, ‘s Hertogenbosch, The Netherlands) daily for 10 days. At the end of the experiment, mice were anaesthesized by intraperitoneal injection with Hypnorm (fentanyl/fluanisone, 1 ml/kg) and Diazepam (10 mg/kg). The gallbladder was canulated and bile was collected for 30 minutes. Subsequently, mice were sacrificed by cardiac puncture and the liver and the small intestine were excised. Feces were collected for 48 hours prior to termination.

### Analytical procedure

Cholesterol was extracted from blood spots and mass fragments m/z 458-165 were analyzed by gas chromatography/mass spectrometry (GC/MS) [20]. Biliary phospholipid concentrations were determined as described previously [20]. Biliary bile acids were determined by an enzymatic fluorimetric assay [26]. Cholesterol in plasma and bile, fecal cholesterol and its derivatives (also called neutral sterols) and biliary bile acid species were determined by gas chromatography [20]. Hepatic lipids were extracted according to Bligh & Dyer [27]. Hepatic triglyceride and cholesterol levels and plasma triglycerides were determined using commercially available kits (Roche Diagnostics, Mannheim, Germany and DiaSys Diagnostic Systems, Holzheim, Germany).

### RNA isolation and measurement of mRNA levels by quantitative real-time PCR

RNA isolation, cDNA synthesis, and real-time quantitative PCR were performed as described previously [7]. PCR results of liver and intestine were normalized to *36B4 mRNA* levels. Primer and probe sequences for the genes tested may be found elsewhere (www.labpediatricsrug.nl).

### Western blotting for Abcg5 protein expression

Brush border isolation and protein determination was performed as previously described [12].

### Statistics

Data are shown as means ± SD. Statistical analysis was assessed by using Kruskal-Wallis H test followed by Conover post-hoc comparisons using the Brightstat [28]. Levels of significance were set at p<0.05.

## Supporting Information

Table S1 **Composition of the semi-synthetic diet.** ^1^Vitamin mix: vitamine A 18.0 IU/g; vitamine D 2.0 IU/g; vitamine D3 2.0 IU/g; vitamine E 62.67 mg/kg; vitamine K3 10.0 mg/kg; vitamine B1 20.01 mg/kg, vitamine B2 11.56 mg/kg; vitamine B6 15.33 mg/kg; Niacin 39.20 mg/kg; pantothenic acid 15.90 mg/kg; vitamine B12 50 µg/kg; folic acid 7.84 mg/kg. ^2^Mineral mix (g/kg): calcium hydrogenphosphate 13.0; calcium carbonate 10; potassium hydrogenphosphate 7.0; potassium chloryde 7.0; sodium chloryde 3.0; magnessium sulphate 4.0; magnesium oxide 2.0; trace elements mix 2.5. ^3^Plant sterol fatty esters mix were mainly β-sitosterol, campesterol and β-sitostanol (69%, 15.7% and 15.7%, respectively).
(DOC)

Table S2 **Intestinal and hepatic gene expression levels in wild-type and** ***Abcg5-/-*** **mice fed control or plant sterol diet.** mRNA was prepared for individual mice and data are presented as means ± SD. Expression values are normalized to mRNA expression of 36B4 and expression in wild-type mice fed control diet was set at 1.00. *p<0.05 control vs plant sterol fed mice; #p<0.05 wild-type vs Abcg5-/- mice with fed the same diet.
(DOC)

## References

[1] Wu T, Fu J, Yang Y, Zhang L, Han J (2009). The effects of phytosterols/stanols on blood lipid profiles: a systematic review with meta-analysis.. Asia Pac J Clin Nutr.

[2] Jones PJ, AbuMweis SS (2009). Phytosterols as functional food ingredients: linkages to cardiovascular disease and cancer.. Curr Opin Clin Nutr Metab Care.

[3] Calpe-Berdiel L, Escola-Gil JC, Blanco-Vaca F (2009). New insights into the molecular actions of plant sterols and stanols in cholesterol metabolism.. Atherosclerosis.

[4] Kaneko E, Matsuda M, Yamada Y, Tachibana Y, Shimomura I (2003). Induction of intestinal ATP-binding cassette transporters by a phytosterol-derived liver X receptor agonist.. J Biol Chem.

[5] Plat J, Mensink RP (2002). Increased intestinal ABCA1 expression contributes to the decrease in cholesterol absorption after plant stanol consumption.. FASEB J.

[6] Plat J, Nichols JA, Mensink RP (2005). Plant sterols and stanols: effects on mixed micellar composition and LXR (target gene) activation.. J Lipid Res.

[7] Plosch T, Kok T, Bloks VW, Smit MJ, Havinga R (2002). Increased hepatobiliary and fecal cholesterol excretion upon activation of the liver X receptor is independent of ABCA1.. J Biol Chem.

[8] Field FJ, Born E, Mathur SN (1997). Effect of micellar beta-sitosterol on cholesterol metabolism in CaCo-2 cells.. J Lipid Res.

[9] Temel RE, Gebre AK, Parks JS, Rudel LL (2003). Compared with Acyl-CoA:cholesterol O-acyltransferase (ACAT) 1 and lecithin:cholesterol acyltransferase, ACAT2 displays the greatest capacity to differentiate cholesterol from sitosterol.. J Biol Chem.

[10] Calpe-Berdiel L, Escola-Gil JC, Julve J, Zapico-Muniz E, Canals F (2007). Differential intestinal mucosal protein expression in hypercholesterolemic mice fed a phytosterol-enriched diet.. Proteomics.

[11] van der Velde AE, Brufau G, Groen AK (2010). Transintestinal cholesterol efflux.. Curr Opin Lipidol.

[12] Kruit JK, Plosch T, Havinga R, Boverhof R, Groot PH (2005). Increased fecal neutral sterol loss upon liver X receptor activation is independent of biliary sterol secretion in mice.. Gastroenterology.

[13] Temel RE, Tang W, Ma Y, Rudel LL, Willingham MC (2007). Hepatic Niemann-Pick C1-like 1 regulates biliary cholesterol concentration and is a target of ezetimibe.. J Clin Invest.

[14] Brown JM, Bell TA, Alger HM, Sawyer JK, Smith TL (2008). Targeted depletion of hepatic ACAT2-driven cholesterol esterification reveals a non-biliary route for fecal neutral sterol loss.. J Biol Chem.

[15] Temel RE, Sawyer JK, Yu L, Lord C, Degirolamo C (2010). Biliary sterol secretion is not required for macrophage reverse cholesterol transport.. Cell Metab.

[16] Plosch T, Kruit JK, Bloks VW, Huijkman NCA, Havinga R (2006). Reduction of cholesterol absorption by dietary plant sterols and stanols in mice is independent of the Abcg5/8 transporter.. J Nutr.

[17] Xie C, Turley SD, Dietschy JM (2009). ABCA1 plays no role in the centripetal movement of cholesterol from peripheral tissues to the liver and intestine in the mouse.. J Lipid Res.

[18] Drobnik W, Lindenthal B, Lieser B, Ritter M, Christiansen WT (2001). ATP-binding cassette transporter A1 (ABCA1) affects total body sterol metabolism.. Gastroenterology.

[19] Groen AK, Bloks VW, Bandsma RH, Ottenhoff R, Chimini G (2001). Hepatobiliary cholesterol transport is not impaired in Abca1-null mice lacking HDL.. J Clin Invest.

[20] van der Veen JN, van Dijk TH, Vrins CL, van Meer H, Havinga R (2009). Activation of the liver X receptor stimulates trans-intestinal excretion of plasma cholesterol.. J Biol Chem.

[21] Yu L, Li-Hawkins J, Hammer RE, Berge KE, Horton JD (2002). Overexpression of ABCG5 and ABCG8 promotes biliary cholesterol secretion and reduces fractional absorption of dietary cholesterol.. J Clin Invest.

[22] Langheim S, Yu L, Von BK, Lutjohann D, Xu F (2005). ABCG5 and ABCG8 require MDR2 for secretion of cholesterol into bile.. J Lipid Res.

[23] Yu L, Hammer RE, Li-Hawkins J, Von BK, Lutjohann D (2002). Disruption of Abcg5 and Abcg8 in mice reveals their crucial role in biliary cholesterol secretion.. Proc Natl Acad Sci U S A.

[24] Plosch T, Bloks VW, Terasawa Y, Berdy S, Siegler K (2004). Sitosterolemia in ABC-transporter G5-deficient mice is aggravated on activation of the liver-X receptor.. Gastroenterology.

[25] van der Velde AE, Vrins CL, van den Oever K, Kunne C, Oude Elferink RP (2007). Direct intestinal cholesterol secretion contributes significantly to total fecal neutral sterol excretion in mice.. Gastroenterology.

[26] Murphy GM, Billing BH, Baron DN (1970). A fluorimetric and enzymatic method for the estimation of serum total bile acids.. J Clin Pathol.

[27] Bligh EG, Dyer WJ (1959). A rapid method of total lipid extraction and purification.. Can J Biochem Physiol.

[28] Stricker D (2008). BrightStat.com: free statistics online.. Comput Methods Programs Biomed.

[29] Mensink RP, de Jong A, Lutjohann D, Haenen GR, Plat J (2010). Plant stanols dose-dependently decrease LDL-cholesterol concentrations, but not cholesterol-standardized fat-soluble antioxidant concentrations, at intakes up to 9 g/d.. Am J Clin Nutr.

[30] Ferezou J, Coste T, Chevallier F (1981). Origins of neutral sterols in human feces studied by stable isotope labeling (D and 13C). Existence of an external secretion of cholesterol.. Digestion.

[31] Yu L, York J, Von BK, Lutjohann D, Cohen JC (2003). Stimulation of cholesterol excretion by the liver X receptor agonist requires ATP-binding cassette transporters G5 and G8.. J Biol Chem.

[32] Calpe-Berdiel L, Rotllan N, Fievet C, Roig R, Blanco-Vaca F (2008). Liver X receptor-mediated activation of reverse cholesterol transport from macrophages to feces in vivo requires ABCG5/G8.. J Lipid Res.

[33] van der Velde AE, Vrins CL, van den Oever K, Seemann I, Oude Elferink RP (2008). Regulation of direct transintestinal cholesterol excretion in mice.. Am J Physiol Gastrointest Liver Physiol.

[34] Vrins CL, van der Velde AE, van den Oever K, Levels JH, Huet S (2009). PPARd activation leads to increased trans intestinal cholesterol efflux.. J Lipid Res.

