# Supplementary material for: A Reappraisal of the Mechanism by Which Plant Sterols Promote Neutral Sterol Loss in Mice
Source: PLoS One. 2011 Jun 30;6(6):e21576. doi: 10.1371/journal.pone.0021576 (PMC3128081; doi:10.1371/journal.pone.0021576)
Supplement: Table S1 — Composition of the semi-synthetic diet. 1Vitamin mix: vitamine A 18.0 IU/g; vitamine D 2.0 IU/g; vitamine D3 2.0 IU/g; vitamine E 62.67 mg/kg; vitamine K3 10.0 mg/kg; vitamine B1 20.01 mg/kg, vitamine B2 11.56 mg/kg; vitamine B6 15.33 mg/kg; Niacin 39.20 mg/kg; pantothenic acid 15.90 mg/kg; vitamine B12 50 µg/kg; folic acid 7.84 mg/kg. 2Mineral mix (g/kg): calcium hydrogenphosphate 13.0; calcium carbonate 10; potassium hydrogenphosphate 7.0; potassium chloryde 7.0; sodium chloryde 3.0; magnessium sulphate 4.0; magnesium oxide 2.0; trace elements mix 2.5. 3Plant sterol fatty esters mix were mainly β-sitosterol, campesterol and β-sitostanol (69%, 15.7% and 15.7%, respectively). (DOC) [file pone.0021576.s001.doc]

|  | **Control** | **1% PS** | **2% PS** | **4% PS** | **8% PS** |
| --- | --- | --- | --- | --- | --- |
|  | % | | | | |
| **Vitamin mix^1^** | 0.25 | | | | |
| **Mineral mix^2^** | 4.85 | | | | |
| **Casein** | 20.00 | | | | |
| **Corn starch** | 54.30 | | | | |
| **Cellulose** | 5.00 | | | | |
| **Soybean oil** | 2.00 | | | | |
| **Coconut fat** | 3.00 | | | | |
| **Sucrose** | 10.00 | | | | |
| **DL Methionine** | 0.20 | | | | |
| **Choline Cl** | 0.40 | | | | |
| **Cholesterol** |  |  | 0.008 |  |  |
| **Plant sterol esters^3^** | - | 1.00 | 2.00 | 4.00 | 8.00 |

^1^Vitamin mix: vitamine A 18.0 IU/g; vitamine D 2.0 IU/g; vitamine D3 2.0 IU/g; vitamine E 62.67 mg/kg; vitamine K3 10.0 mg/kg; vitamine B1 20.01 mg/kg, vitamine B2 11.56 mg/kg; vitamine B6 15.33 mg/kg; Niacin 39.20 mg/kg; pantothenic acid 15.90 mg/kg; vitamine B12 50 μg/kg; folic acid 7.84 mg/kg. ^2^Mineral mix (g/kg): calcium hydrogenphosphate 13.0; calcium carbonate 10; potassium hydrogenphosphate 7.0; potassium chloryde 7.0; sodium chloryde 3.0; magnessium sulphate 4.0; magnesium oxide 2.0; trace elements mix 2.5. ^3^ Plant sterol fatty esters mix were mainly β-sitosterol, campesterol and β-sitostanol (69%, 15.7% and 15.7%, respectively).
