# Supplementary material for: A Reappraisal of the Mechanism by Which Plant Sterols Promote Neutral Sterol Loss in Mice
Source: PLoS One. 2011 Jun 30;6(6):e21576. doi: 10.1371/journal.pone.0021576 (PMC3128081; doi:10.1371/journal.pone.0021576)
Supplement: Table S2 — Intestinal and hepatic gene expression levels in wild-type and Abcg5-/- mice fed control or plant sterol diet. mRNA was prepared for individual mice and data are presented as means ± SD. Expression values are normalized to mRNA expression of 36B4 and expression in wild-type mice fed control diet was set at 1.00. *p<0.05 control vs plant sterol fed mice; #p<0.05 wild-type vs Abcg5-/- mice with fed the same diet. (DOC) [file pone.0021576.s002.doc]

|  | **Wild-type** | | | | |  | ***Abcg5^-/-^*** | |
| --- | --- | --- | --- | --- | --- | --- | --- | --- |
|  | **0% PS** | **1% PS** | **2% PS** | **4% PS** | **8% PS** |  | **0% PS** | **8% PS** |
| **Liver** |  |  |  |  |  |  |  |  |
| ***Abcg5*** | 1.00 ± 0.50 | 0.20 ± 0.04* | 0.52 ± 0.35* | 1.08 ± 0.19 | 1.11 ± 0.35 |  | 0.04 ± 0.02^#^ | 0.08 ± 0.03^#^ |
| ***Abcg8*** | 1.00 ± 0.33 | 0.78 ± 0.18* | 0.70 ± 0.22* | 0.80 ± 0.18 | 0.66 ± 0.23* |  | 0.63 ± 0.28 | 0.56 ± 0.40 |
| ***Abca1*** | 1.00 ± 0.22 | 0.85 ± 0.15 | 0.91 ± 0.26 | 1.08 ± 0.51 | 0.95 ± 0.18 |  | 1.24 ± 0.26 | 1.24 ± 0.65 |
| ***Sr-b1*** | 1.00 ± 0.24 | 0.66 ± 0.09 | 0.62 ± 0.10 | 0.84 ± 0.29 | 0.88 ± 0.35 |  | 1.02 ± 0.44 | 0.46 ± 0.33 |
| ***Hmgcr*** | 1.00 ± 0.47 | 1.41 ± 0.29 | 1.73 ± 0.59 | 1.93 ± 0.36* | 2.46 ± 0.48* |  | 0.96 ± 0.69 | 1.15 ± 0.94 |
| **Ileum** |  |  |  |  |  |  |  |  |
| ***Abcg5*** | 1.00 ± 0.42 | 1.05 ± 0.54 | 0.61 ± 0.14* | 0.66 ± 0.18* | 0.72 ± 0.13* |  | 0.06 ± 0.02^#^ | 0.03 ± 0.02^#^ |
| ***Abcg8*** | 1.00 ± 0.27 | 1.52 ± 1.09 | 0.87 ± 0.21 | 1.02 ± 0.34 | 0.60 ± 0.12* |  | 0.21 ± 0.04^#^ | 0.12 ± 0.01^#^ |
| ***Npc1l1*** | 1.00 ± 0.16 | 2.48 ± 2.12* | 0.97 ± 0.13 | 1.23 ± 0.47 | 1.02 ± 0.24 |  | 1.29 ± 0.24^#^ | 0.61 ± 0.17*^#^ |
| ***Abca1*** | 1.00 ± 0.62 | 1.29 ± 1.40 | 0.48 ± 0.13* | 0.68 ± 0.41 | 0.58 ± 0.28 |  | 1.17 ± 0.42 | 1.42 ± 1.92^#^ |
| ***Sr-b1*** | 1.00 ± 0.35 | 1.41 ± 0.68 | 1.25 ± 0.19 | 1.36 ± 0.41 | 1.48 ± 0.90 |  | 1.33 ± 0.87 | 0.63 ± 0.80 |
| ***Hmgcr*** | 1.00 ± 0.26 | 1.68 ± 0.94 | 1.53 ± 0.12* | 1.51 ± 0.24* | 1.53 ± 0.52* |  | 1.30 ± 0.38 | 1.36 ± 0.43 |

mRNA was prepared for individual mice and data are presented as means ± SD. Expression values are normalized to mRNA expression of 36B4 and expression in wild-type mice fed control diet was set at 1.00. *p<0.05 control vs plant sterol fed mice; #p<0.05 wild-type vs Abcg5-/- mice with fed the same diet.
